# Revisiting Echocardiographic Ranges of Left Ventricular End‐Diastolic Volume Index: An Analysis of the Discrepancies Between the 2006 and the 2015 Recommendation for Chamber Quantification Guidelines

**DOI:** 10.1002/clc.70003

**Published:** 2024-08-28

**Authors:** Parisa Fallahtafti, Reza Bahramrafiee, Roya Sattarzadeh Badkoubeh, Akram Sardari, Mohammad Reza Eftekhari, Babak Geraiely, Farnoosh Larti

**Affiliations:** ^1^ Cardiology Department, Imam Khomeini Hospital Complex Tehran University of Medical Sciences Tehran Iran

**Keywords:** chamber quantification guidelines, discrepancy, LV end‐diastolic volume index, LVEDVi, reclassification

## Abstract

**Background:**

Indexed left ventricular end‐diastolic volume (LVEDVi) is a left ventricle (LV) size marker. The “Recommendations for Chamber Quantification” guideline was published in 2006 and updated in 2015. Although the previous guideline maintained uniform cutoff points for both men and women, the latest revision introduced new thresholds that vary between genders. We evaluated the extent of change in labeled indexed LV diastolic volumes in men and women following the adoption of the 2015 guideline.

**Methods:**

Data were extracted from a web‐based registry from March 2020 to October 2022. LV indexed volume variables were categorized on the basis of the 2006 and 2015 guidelines.

**Results:**

Among the 7598 individuals, the classification of LVEDVi differed in 910 (12.0%) individuals. In 213 (5.5%) female subjects, substantial reclassification (i.e., transitioning from normal to moderate LV enlargement to mild to severe LV enlargement) occurred on the basis of the 2015 guideline. All females classified as having moderately abnormal LVEDVi according to the 2006 guideline were reclassified as having severely abnormal LVEDVi according to the 2015 guideline. Age, LV ejection fraction (LVEF), and significant aortic regurgitation (AR) were common factors contributing to the observed discrepancy in both men and women. Significant mitral regurgitation (MR) and regional or global motion abnormality were correlated with the reclassification of LVEDVi to higher abnormal partitions only in women.

**Conclusion:**

The observed disparities underscore the importance of ongoing dedicated research to reassess the range of indexed echocardiographic parameters, considering various outcomes and differences in countries.

## Graphical abstract

Assessment of the left ventricular end‐diastolic volume index between the 2006 and 2015 guidelines and the magnitude of the observed discrepancy, especially in women, underscores the importance of applying the normal ranges of echocardiographic parameters in different countries.

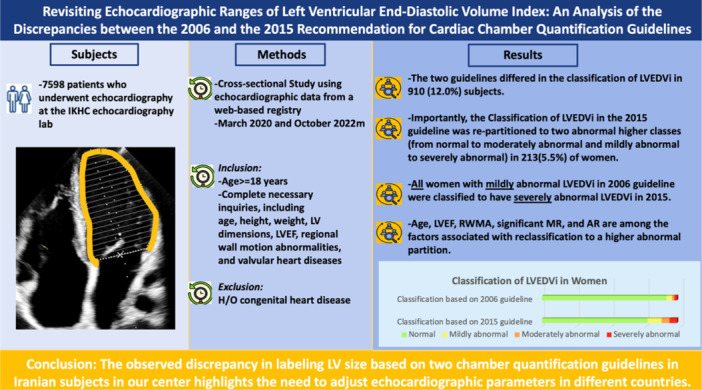

**Abbreviations**
LVE: left ventricular enlargement
LVEDD: left ventricular end‐diastolic diameter
LVEDDi: indexed left ventricular end‐diastolic diameter
LVEDV: left ventricular end‐diastolic volume
LVEDVi: indexed left ventricular end‐diastolic volume

## 1 Introduction

Left ventricular enlargement (LVE) manifests as increased dimensions of the left ventricle, either as a physiological adaptation or due to cardiac pathologies. The diagnosis of LVE typically involves imaging modalities such as echocardiography and cardiac magnetic resonance (CMR) imaging. Although CMR is considered the gold standard, echocardiography is widely utilized and valuable for assessing heart structure and function [1, 2]. Various guidelines, including the “Recommendations for Cardiac Chamber Quantification by Echocardiography in Adults,” advocate measuring left ventricular (LV) size, diameter, and volume. The internal diameter of the LV at end‐diastole (LVEDD) and end‐systole (LVESD) is commonly measured [1], and these parameters are often combined with LV end‐diastolic volume (LVEDV) and LV end‐systolic volume (LVESV), indexed to the body surface area (BSA) [1]. However, the correlation between LVEDD and LVEDV is sometimes linear, potentially leading to misclassification of LVE [3, 4].

In managing valvular heart disease, LV diameter holds significance for surgical decision‐making in aortic and mitral insufficiency, as per the 2020 ACC/AHA and 2021 ESC/EACTS guidelines [5, 6]. Yet, no recommendations were proposed based on volumetric LV analysis in the recent valvular guidelines [4]. Given that LVEDD may underestimate LV volume in remodeled LV, it is crucial to recognize that a simple measurement of LVEDD is not interchangeable with LV volume [7].

Echocardiographic reference values for cardiac parameters were initially published in 2006 and updated in 2015 by the American Society of Echocardiography (ASE) in collaboration with the European Association of Echocardiography. Although these guidelines are essential for quantitative echocardiography, they may only partially represent the diversity in the world population. Evidence suggests significant variations in LV size parameters across different countries [8].

These measurements are indexed to the BSA to correct LV volume and dimension for body size [9, 10]. However, changes in cutoff values for indexed LV volumes between the 2006 and 2015 guidelines, particularly for women, imply that even after indexing to BSA, the classification of LV enlargement may differ on the basis of gender, potentially leading to the relabeling of individuals and influencing subsequent diagnostic workup [1].

In our echocardiography lab, we encountered serial echocardiography reports in women with normal echocardiography results between 2006 and 2015 who fell into different LV‐size partitions with no identifiable cardiovascular disease. This observation revealed discrepancies in LV size labeling that could have resulted in unnecessary workups, which prompted this study. The study's primary aim is to evaluate the extent of change in labeled indexed LV diastolic volumes in men and women following the adoption of the 2015 guidelines and identify potential contributing factors to this change.

## 2 Materials and Methods

### 2.1 Study Participants

This cross‐sectional study utilized echocardiographic data from a web‐based registry comprising 515 echocardiographic and clinical variables. The study population consisted of patients who underwent echocardiography at the Imam Khomeini Hospital Complex (IKHC) echocardiography lab between March 2020 and October 2022. Demographic data, including age, height, weight, and BSA, were extracted from the registry database. Detailed information on left ventricular end‐diastolic diameter (LVEDD), LVEDV, left ventricular end‐systolic diameter (LVESD), BSA, left ventricular ejection fraction (LVEF), regional wall motion abnormalities, and valvular heart diseases was available for the included subjects. Past medical history, encompassing congenital heart disease, valvular heart disease, ischemic heart disease, and any previous cardiac surgery, was also collected.

Given the retrospective nature of our methodology, an informed consent waiver was obtained from the ethics committee. The primary exclusion criteria were a history of congenital heart disease and incomplete necessary inquiries. The research ethics committees of the Tehran University of Medical Sciences approved the study (IR.TUMS.IKHC.REC.1401.308), and all investigations were conducted in accordance with the 1975 Declaration of Helsinki.

### 2.2 Echocardiography

The data were sourced from the web‐based echocardiographic data registry of IKHC, comprising echocardiographic data from 7598 patients. Within our lab, the data in the web‐based registry originate exclusively from echocardiography examinations conducted by attending physicians, precisely four expert cardiologists with fellowships in echocardiography. Echocardiograms were performed with superimposed electrocardiogram (ECG) tracing, with end‐diastole defined at the peak R wave of the ECG. The measured LVEDD, LVESD, LVEDV, and left ventricular end‐systolic volume (LVESV) were automatically indexed by BSA in our web‐based registry. Assessment of wall motion abnormality and valvular heart disease, including aortic regurgitation (AR), aortic stenosis (AS), and mitral regurgitation (MR), was conducted according to the latest available guidelines. Moderate or more mitral or AR was defined as significant.

**Figure 1:**
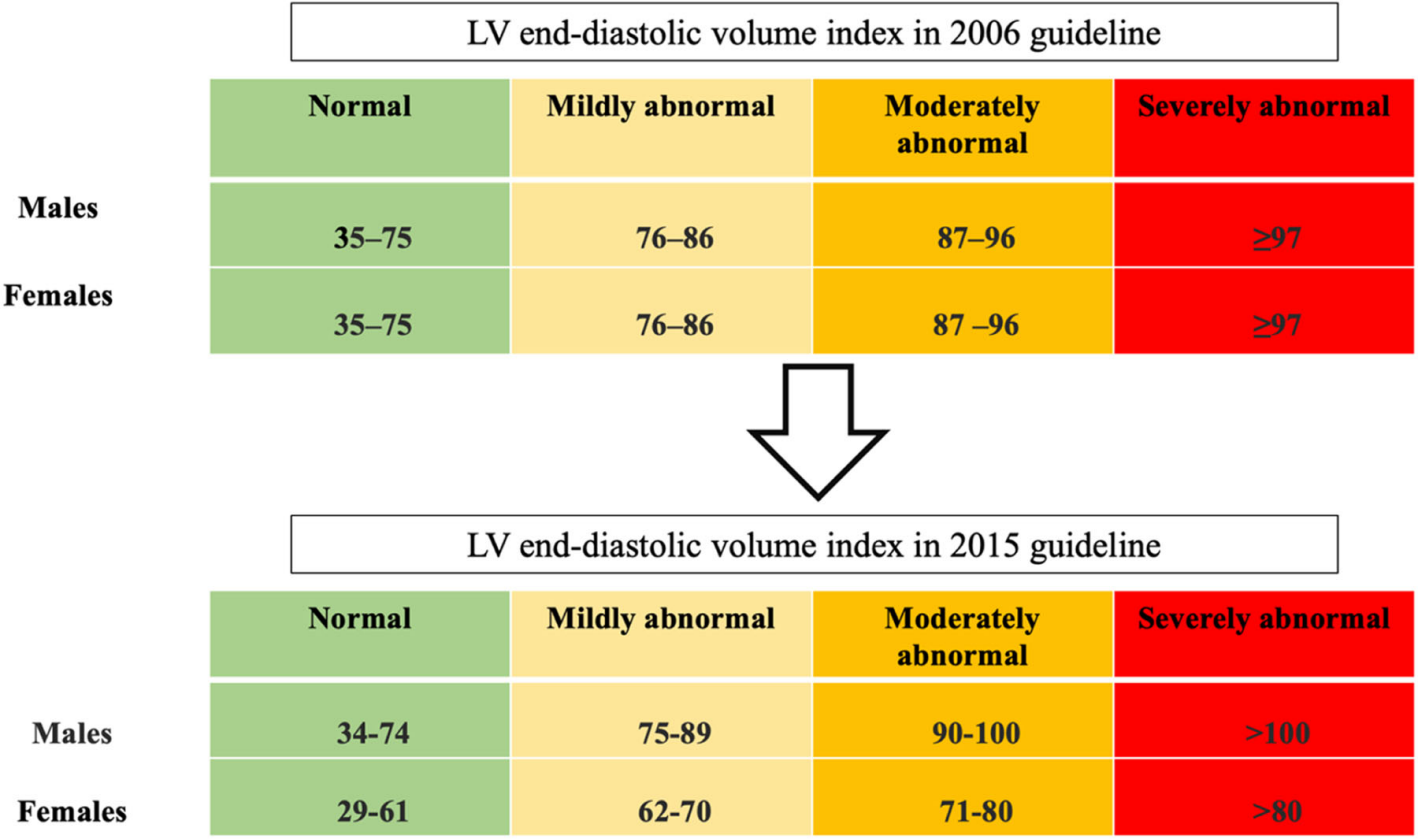
Reference ranges and partition cutoff values for 2D echocardiography‐derived LV end‐diastolic volume index in 2006 and 2015 guidelines in men and women.

Chamber quantification by 2D echocardiography was performed in accordance with the latest American Society of Echocardiography (ASE) guideline. Figure 1 illustrates the differences between the 2006 and 2015 guidelines for indexed left ventricular end‐diastolic volume (LVEDVi) in men and women. For clarity, we will refer to the “Guideline of Recommendations for Chamber Quantification 2006” as the 2006 guideline and “Recommendations for Cardiac Chamber Quantification by Echocardiography in Adults 2015” as the 2015 guideline.

### 2.3 Statistical Analysis

The results of quantitative variables were reported as mean and standard deviation (SD), whereas qualitative variables were reported as counts and percentages. Agreement between the 2006 and 2015 guidelines results was assessed using the *κ*‐coefficient and cross tables. Logistic regression analysis was used to investigate factors influencing differences between the guidelines. An independent *t*‐test was utilized to compare means in two‐group variable analysis, whereas analysis of variance (ANOVA) was applied for groups with more than two modes. The chi‐squared test and Fisher's exact test were used to compare qualitative variables.

Statistical analysis was conducted using SPSS version 25, with a significance level set at 0.05.

## 3 Results

### 3.1 Demographics

In our study, 7598 individuals were included, with 3875 (51%) females. The mean age was 49.0 years (SD = 16.0) for females and 52.1 years (SD = 16.2) for males. The age range spanned from 18 to 98 years. Regarding anthropometric measurements, the mean weight was 69.1 kg (SD = 14.3) for females and 76.5 kg (SD = 15.0) for males. The mean height was 160.7 cm (SD = 7.7) for females and 172.3 cm (SD = 8.9) for males. In terms of LV function, the mean ejection fraction (EF) was 44.9% (SD = 12.1) for males, 48.4% (SD = 9.7) for females, and 46.7% (SD = 11.1) for the total population.

### 3.2 LVEDVi

According to the 2006 and 2015 guidelines, we separately classified the measured LVEDVi to BSA into four groups: reference (normal) range, mildly abnormal, moderately abnormal, and severely abnormal LVEDVi (Table 1).

**Table 1:** Agreement of LVEDVi partitions between 2006 and 2015 guidelines in the total population.

		2015 Guideline				
		Normal range	Mildly abnormal	Moderately abnormal	Severely abnormal	Total
2006 Guideline	Normal range	5949 (91.2%)	433 (6.6%)	141 (2.2%)	0 (0.0%)	6523 (100%)
	Mildly abnormal	0 (0.0%)	278 (63.6%)	87 (19.9%)	72 (16.5%)	437 (100%)
	Moderately abnormal	0 (0.0%)	63 (26.1%)	110 (45.6%)	68 (28.2%)	241 (100%)
	Severely abnormal	0 (0.0%)	0 (0.0%)	46 (11.6%)	351 (88.4%)	397 (100%)
	Total	5949	774	384	491	7598

*Note:* Shaded boxes show the number and percentage of subjects with the same labeled partition based on 2006 and 2015 guidelines.

### 3.3 Reclassification of LVEDVi

Use of two different guidelines resulted in reclassification of LVEDVi in 910 (12.0%) subjects, with a Cohen's *κ*‐coefficient of 0.622. Among men, the new LVEDVi class differed in 146 (3.9%) subjects, whereas it differed in 764 (19.7%) subjects among women.

In men, under the 2015 guideline, LVEDVi was repartitioned in 109 (2.9%) subjects from moderately abnormal to mildly abnormal and from severely abnormal to moderately abnormal. Conversely, it was repartitioned in 37 (1.0%) subjects from normal to mildly elevated. Notably, the number of men with mildly elevated LVEDVi remained the same in both guidelines (Table 2).

**Table 2:** Agreement of LVEDVi partitions between 2006 and 2015 guidelines in men and women and direction and magnitude of the changes.

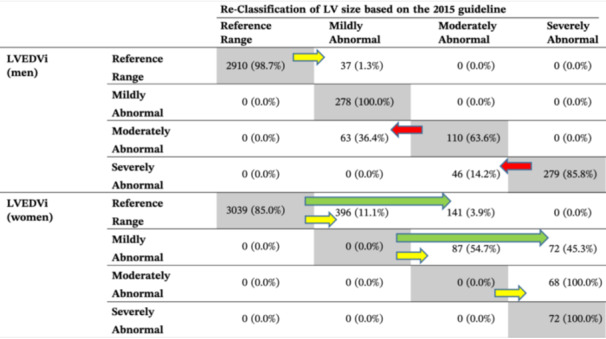

*Note:* The magnitude and direction of LVEDVi reclassification after applying the 2015 guideline are illustrated by arrows. Shaded boxes show the number and percentage of subjects with the same labeled partition based on 2006 and 2015 guidelines. 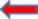: Reclassification to one lower partition; 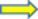: reclassification into one higher abnormal partition; 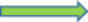: reclassification into two higher abnormal partitions.

As expected, classification under the 2015 guideline was reclassified to a higher abnormal partition in 213 (5.5%) female subjects. Specifically, in 141 (3.6%) cases, LVEDVi changed from normal to moderately abnormal, and in 72 (1.9%) cases, it switched from mildly abnormal to severely abnormal. Notably, women who had moderately abnormal LVEDVi, according to the 2006 guideline, were classified as severely abnormal under the 2015 guideline. Considering the new cutoffs, no downgrading was observed in the reclassification of LVEDVi in females (Table 2).

According to the 2015 guideline, all women previously classified as having mildly abnormal LVEDVi were reclassified as having moderately or severely abnormal LVEDVi. Among these subjects, the mean LVEF of those reclassified as moderately abnormal was 39.6 (SD = 14.1), whereas it was 39.8 (SD = 14.8) in the severely abnormal group. Detailed characteristics of individuals with discrepancies in LVEDVi classification are provided in Supporting Information S1: Table 1.

### 3.4 Factors Associated With the Observed Discrepancies in LVEDVi Reclassification

As the pattern of reclassification of LVEDVi differed between men and women, we evaluated the affecting factors stratified by sex (Table 3). Age, LVEF, and significant AR were common factors contributing to the observed discrepancy between men and women. Younger age and decreasing LVEF are associated with reclassification to a higher abnormal partition. At the same time, significant AR was related to reclassification to higher abnormal partitions in the 2015 guideline. Moreover, height was associated with reclassification of LVEDVi to a lower partition in women. At the same time, significant MR and the presence of regional or global motion abnormality were correlated with the reclassification of LVEDVi to higher abnormal partitions in the 2015 guideline in women. It is noteworthy that significant AR roughly triples the risk of repartitioning.

**Table 3:** Factors related to the observed discrepancy between two LVEDVi classifications in 2005 and 2016 guidelines in men and women.

Factor	*B*	Sig.	Exp (*B*) (95% CI)
Age			
Men	−0.013	0.043	0.987 (0.974–1.000)
Women	−0.013	0.000	0.987 (0.981–0.992)
Weight			
Men	−0.004	0.541	0.996 (0.983–1.009)
Women	−0.001	0.661	0.999 (0.993–1.005)
Height			
Men	−0.018	0.093	0.982 (0.962–1.003)
Women	−0.012	0.034	0.988 (0.977–0.999)
LVEF			
Men	−0.052	0.000	0.949 (0.935–0.964)
Women	−0.054	0.000	0.947 (0.939–0.956)
No wall motion abnormality, no global motion abnormality			
Men	1.051	0.058	2.859 (0.963–8.485)
Women	0.332	0.162	1.394 (0.875–2.220)
Regional motion abnormality or global motion abnormality			
Men	0.766	0.163	2.152 (0.733–6.313)
Women	0.517	0.025	1.677 (1.066–2.639)
Significant MR			
Men	0.351	0.201	1.420 (0.829–2.432)
Women	0.975	0.000	2.651 (2.034–3.455)
Significant AS			
Men	−0.451	0.466	0.637 (0.189–2.141)
Women	0.397	0.230	1.488 (0.778–2.847)
Significant AR			
Men	1.174	0.000	3.235 (1.809–5.783)
Women	1.356	0.000	3.881 (2.691–5.596)

Abbreviations: AR, aortic regurgitation; AS, aortic stenosis; EF, ejection fraction; LV, left ventricle; MR, mitral regurgitation.

The supplementary files provide the same results, focusing on LVEDDi (Supporting Information S1: Tables 2–4).

## 4 Discussion

Measurements of LV internal diameter and volume at LVESD and LVEDD are fundamental indicators of LV cavity size [1]. Alongside LVEF, BSA–indexed LVEDV and LVESV play pivotal roles in clinical decision‐making and predicting outcomes in cardiology [2].

Our study showed that the two guidelines differed in the classification of LVEDVi in 910 (12.0%) subjects, comprising 3.9% of men and 19.7% of women. Applying the 2015 guideline resulted in reclassification of women to two abnormal higher classes (from normal to moderately abnormal and mildly abnormal to severely abnormal) in 213 (5.5%) women. One crucial point is that all women with mildly abnormal LVEDVi in 2006 guidelines were classified to have severely abnormal LVEDVi. The discrepancies in guidelines' reference values for LV dimensions and volume stemmed from changes in cutoff values and study population characteristics. The magnitude of this considerable repartitioning has yet to be studied properly in prospective studies.

Misclassification can significantly impact patient management and health outcomes [1, 3]. For instance, patients classified incorrectly may not receive timely interventions that are critical for their health [4]. On the contrary, the transition of patients to higher abnormal classifications can result in unnecessary diagnostic testing, leading to increased healthcare costs and patient anxiety without a clear clinical benefit. This phenomenon has been documented in studies where patients with mild abnormalities were subjected to extensive workups due to misclassification, ultimately causing more harm than good [11]. The range of LVEDVi underwent significant changes in 2015 compared to the 2006 guideline, particularly in women. The 2006 guideline derived reference values for LV linear dimensions from a cohort of 510 White, African American, and American Indian adults without recognized cardiovascular disease, hypertension, or obesity. LV volume references were obtained from 52 healthy adults, 29 men and 23 women. The evaluated normal population showed a weak correlation between BSA and LVEDV, with men having larger LV volumes [5]. In contrast, the 2015 guideline reference values were obtained from a meta‐analysis of population‐based studies across seven databases [6, 7, 8, 9, 10, 12]. Although these values represent a diverse study population, predominantly from the United States or Europe, they may not universally apply due to limitations in racial and ethnic diversity and the heterogeneous methodology of studies and included echocardiographic data sets.

The current LV enlargement cutoffs lack correlation with observed outcomes, as the abnormality classification relies on normal reference values using SD rather than outcomes. Population‐specific adjustment of echocardiographic parameters is crucial, as demonstrated by the significant intercountry variation in LV size‐related parameters observed in a study by the World Alliance Societies of Echocardiography (WASE). Discrepancies in LV dimensions and volume reference values between guidelines stem from changes in cutoff values and study population characteristics. For instance, a study on Iranian men and women reported normal ranges of LV internal diameter at end‐diastole (LVIDd) and LVEDV differing from those suggested by chamber quantification guidelines, emphasizing the role of nationality in indexed values [12].

In our study, factors such as sex, height, weight, LVEF, and the presence of MR or AR influenced the discrepancy in guideline classification of indexed LVEDD. Age, LVEF, height, significant MR, and significant AR also correlated with differences in the indexed LVEDV classification between the two guidelines. Consistent with our findings, a study by Alizadehasl et al. on 2229 Iranian subjects demonstrated variations in echocardiographic parameters with age, sex, and race [13].

## 5 Strengths and Limitations

The current study demonstrated several strengths. The large‐scale cross‐sectional design allowed for a robust comparison of LV dimension classification according to the two latest Chamber Quantification guidelines. However, several limitations should be acknowledged. First, the study was conducted at a single center over a relatively limited time period. Second, due to potential errors in measurement of end‐systolic–related variables, the analysis did not include end‐systolic volume measurements. Notably, the current study did not compare clinical outcomes between the two guideline classifications, a crucial limitation that should be addressed in future research. Despite including many factors in the study, there may be potential confounding variables that should have been accounted for.

## 6 Conclusion

The observed discrepancy in labeling LV size on the basis of two chamber quantification guidelines in Iranian subjects in our study highlights the need to adjust echocardiographic parameters to different countries and, importantly, the need for careful supervision by committee members who manage projects establishing standard data and clear protocols for obtaining and measuring images. More population‐based cohort studies should be conducted to define the reference ranges for each sex.

## Conflicts of Interest

The authors declare no conflicts of interest.

## Supporting information

Supporting information.

## Data Availability

The data that support the findings of this study are available on request from the corresponding author. The data are not publicly available due to privacy or ethical restrictions.
