# Supplementary material for: Revisiting Echocardiographic Ranges of Left Ventricular End‐Diastolic Volume Index: An Analysis of the Discrepancies Between the 2006 and the 2015 Recommendation for Chamber Quantification Guidelines
Source: Clin Cardiol. 2024 Aug 28;47(9):e70003. doi: 10.1002/clc.70003 (PMC11350272; doi:10.1002/clc.70003)
Supplement: Supplementary file 1 — Supporting information. [file CLC-47-e70003-s001.docx]

**Supplementary Materials**

**Supplementary table 1. Characteristic of individuals with the discrepancy in the classification of LVEDVi**

**Supplementary Table 2. Agreement of LVEDDi partitions between 2006 and 2015 guidelines in the total population**

**Supplementary Table 3. Agreement of LVEDDi partitions between 2006 and 2015 guidelines in men and women**

**Supplementary table 4. Factors related to the discrepancy between two LVEDDi classifications in guidelines of Chamber Quantification 2006 and Recommendations for Cardiac Chamber Quantification by Echocardiography in Adults 2015**

# **Supplementary table 1.** Characteristic of individuals with the discrepancy in the classification of LVEDVi

| **LVEDVi based on 2006 guideline** | **LVEDVi based on 20015 guideline** | **Sex** | **Age** | **Weight** | **Height** | **EF** | **No wall motion abnormality** | **Regional motion abnormality** | **Global motion abnormality** | **No MR or mild MR** | **Moderate MR** | **Severe MR** | **Significant AS** | **No AR or mild AR** | **Moderate AR** | **Severe AR** |
| --- | --- | --- | --- | --- | --- | --- | --- | --- | --- | --- | --- | --- | --- | --- | --- | --- |
| Normal | Normal | Male | 51.9 ± 16.3 | 76.9 ± 15.1 | 172.6 ± 8.9 | 47.5 ± 9.4 | 58.8% | 38.6% | 2.6% | 91.5% | 6.9% | 1.6% | 2.8% | 93.9% | 5.6% | 0.5% |
|  | Mild |  | 54.0 ± 16.4 | 74.9 ± 16.9 | 170.7 ± 6.8 | 41.1 ± 12.7 | 54.1% | 40.5% | 5.4% | 78.4% | 16.2% | 5.4% | 0% | 83.8% | 16.2% | 0% |
| Moderate | Mild | Male | 51.0 ± 16.4 | 70.7 ± 13.4 | 170.8 ± 8.6 | 37.9 ± 15.2 | 54.0% | 44.4% | 1.6% | 81.0% | 15.9% | 3.1% | 3.2% | 85.7% | 1.6% | 12.7% |
|  | Moderate |  | 52.8 ± 16.9 | 76.0 ± 13.5 | 172.0 ± 8.2 | 36.1 ± 14.6 | 43.6% | 48.2% | 8.2% | 77.3% | 12.7% | 10.0% | 3.6% | 77.3% | 11.8% | 10.9% |
| Severe | Moderate | Male | 51.3 ± 18.8 | 78.9 ± 14.5 | 171.5 ± 16.5 | 36.8 ± 16.6 | 48.9% | 44.4% | 6.7% | 65.2% | 10.9% | 23.9% | 3.2% | 76.1% | 10.9% | 13.0% |
|  | Severe |  | 53.0 ± 14.5 | 74.8 ± 15.0 | 171.5 ± 8.5 | 28.9 ± 15.6 | 41.9% | 44.8% | 13.3% | 52.0% | 26.5% | 21.5% | 5.7% | 74.6% | 6.1% | 19.3% |
| Normal | Normal | Female | 49.1 ± 16.0 | 69.5 ± 14.1 | 160.9 ± 7.4 | 50.2 ± 7.5 | 74.3% | 23.7% | 2.0% | 89.8% | 8.9% | 1.3% | 1.4% | 94.2% | 5.7% | 0.1% |
|  | Mild |  | 48.5 ± 16.2 | 68.5 ± 15.2 | 160.0 ± 9.2 | 45.2 ± 10.9 | 61.9% | 32.6% | 5.5% | 80.3% | 13.9% | 5.8% | 2.8% | 88.4% | 10.6% | 1.0% |
|  | Moderate |  | 50.5 ± 16.2 | 67.5 ± 12.5 | 160.3 ± 7.3 | 45.1 ± 11.5 | 53.9% | 42.6% | 3.5% | 70.9% | 19.1% | 10.0% | 1.4% | 82.3% | 12.7% | 5.0% |
| Mild | Moderate | Female | 48.8 ± 14.7 | 68.4 ± 13.6 | 160.8 ± 6.8 | 39.6 ± 14.1 | 56.3% | 31.1% | 12.6% | 69.0% | 18.4% | 12.6% | 1.1% | 89.7% | 6.9% | 3.4% |
|  | Severe |  | 46.0 ± 16.2 | 67.9 ± 17.4 | 159.1 ± 7.3 | 39.8 ± 14.8 | 59.7% | 30.6% | 9.7% | 63.9% | 29.2% | 6.9% | 1.4% | 86.1% | 8.3% | 5.6% |
| Moderate | Severe | Female | 47.1 ± 16.6 | 65.9 ±16.1 | 160.2 ± 8.1 | 38.1 ± 14.2 | 57.4% | 39.7% | 2.9% |  |  |  |  |  |  |  |
| Normal | Normal | Male  and  female | 50.4 ± 16.2 | 73.1 ± 15.1 | 166.7 ± 10.0 | 48.9 ± 8.6 | 66.7% | 31.0% | 2.3% | 90.6% | 7.9% | 1.5% | 2.1% | 94.0% | 5.7% | 0.3% |
|  | Mild |  | 49.0 ± 16.2 | 69.0 ± 15.4 | 160.9 ± 9.5 | 44.9 ± 11.1 | 61.2% | 33.3% | 5.5% | 80.1% | 14.1% | 5.8% | 2.5% | 88.0% | 11.1% | 0.9% |
|  | Moderate |  | 50.5 ± 16.2 | 67.5 ± 12.5 | 160.3 ± 7.3 | 45.1 ± 11.5 | 53.9% | 42.6% | 3.5% | 70.9% | 19.1% | 10.0% | 1.4% | 82.3% | 12.7% | 5.0% |
| Mild | Mild | Male and female | 53.5 ± 16.0 | 75.3 ± 15.1 | 170.7 ± 8.4 | 40.2 ± 13.8 | 48.6% | 47.5% | 4.0% | 74.1% | 14.7% | 11.2% | 3.2% | 82.4% | 12.9% | 4.7% |
|  | Moderate |  | 48.8 ± 14.7 | 68.4 ± 13.6 | 160.8 ± 6.8 | 39.6 ± 14.1 | 56.3% | 31.0% | 12.6% | 69.0% | 18.4% | 12.6% | 1.1% | 89.7% | 6.9% | 3.4% |
|  | Severe |  | 46.0 ± 16.2 | 67.9 ± 17.4 | 159.1 ± 7.3 | 39.8 ± 14.8 | 59.7% | 12.6% | 9.7% | 63.9% | 29.2% | 6.9% | 1.4% | 86.1% | 8.3% | 5.6% |
| Moderate | Mild | Male and female | 51.0 ± 16.4 | 70.7 ± 13.4 | 170.8 ± 8.6 | 37.9 ± 15.2 | 54.0% | 44.4% | 1.6% | 81.0% | 15.9% | 3.1% | 3.2% | 85.7% | 1.6% | 12.7% |
|  | Moderate |  | 52.8 ± 16.9 | 76.0 ± 13.5 | 172.0 ± 8.2 | 36.1 ± 14.6 | 43.6% | 48.2% | 8.2% | 77.3% | 12.7% | 10.0% | 3.6% | 77.3% | 11.8% | 8.8% |
|  | Severe |  | 47.1 ± 16.6 | 65.9 ± 16.1 | 160.2 ± 8.1 | 36.1 ± 14.6 | 57.4% | 39.7% | 2.9% | 64.7% | 22.1% | 13.2% | 2.9% | 83.8% | 8.8% | 7.4% |
| Severe | Moderate | Male and female | 51.3 ± 18.8 | 78.9 ± 14.5 | 171.5 ± 16.5 | 36.8 ± 16.6 | 48.9% | 44.4% | 6.7% | 65.2% | 10.9% | 23.9% | 2.2% | 76.1% | 10.9% | 13.0% |
|  | Severe |  | 52.4 ± 15.2 | 73.0 ± 15.6 | 169.0 ± 10.5 | 28.3 ± 15.4 | 41.3% | 42.5% | 16.% | 53.0% | 25.9% | 21.1% | 4.6% | 74.6% | 7.5% | 17.9% |

AR, aortic regurgitation; AS, aortic stenosis; EF, ejection fraction; LV, left ventricle; LV, left ventricle; MR, mitral regurgitation

# **Supplementary Table 2.** Agreement of LVEDDi partitions between 2006 and 2015 guidelines in the total population

|  |  | 2015 Guideline | | | | |
| --- | --- | --- | --- | --- | --- | --- |
|  | | **Normal**  **Range** | **Mildly Abnormal** | **Moderately Abnormal** | **Severely Abnormal** | **Total** |
| 2006 Guideline | **Normal**  **range**  **N (%)** | 6386 (95.0%) | 335 (5.0%) | 0 (0.0%) | 0 (0.0%) | 6721 (100%) |
|  | **Mildly**  **Abnormal**  **N (%)** | 0 (0.0%) | 393 (85.2%) | 68 (14.8%) | 0 (0.0%) | 461 (100%) |
|  | **Moderately Abnormal N (%)** | 0 (0.0%) | 0 (0.0%) | 143 (69.8%) | 62 (30.2%) | 205 (100%) |
|  | **Severely**  **Abnormal N (%)** | 0 (0.0%) | 0 (0.0%) | 0 (0.0%) | 211 (100%) | 211 (100%) |
|  | **Total** | 6386 | 728 | 211 | 273 | 7598 |

#

| **Re-Classification of LV end-diastolic diameter based on the 2015 guideline** | | | |  |  |  |
| --- | --- | --- | --- | --- | --- | --- |
| **Normal range** | **Mildly Abnormal** | **Moderately Abnormal** | **Severely Abnormal** |  |  |  |
| **Indexed data based on the 2006 guideline** | **LVEDDi**  **(males)** | **Normal**  **range** | 3066 (94.7%) | 173 (5.3%) | 0 (0.0%) | 0 (0.0%) |
|  |  | **Mildly Abnormal** | 0 (0.0%) | 195 (74.1%) | 68 (25.9%) | 0 (0.0%) |
|  |  | **Moderately Abnormal** | 0 (0.0%) | 0 (0.0%) | 53 (58.9%) | 37 (41.1%) |
|  |  | **Severely Abnormal** | 0 (0.0%) | 0 (0.0%) | 0 (0.0%) | 131 (100%) |
|  | **LVEDDi**  **(females)** | **Normal range** | 3320 (95.3%) | 162 (4.7%) | 0 (0.0%) | 0 (0.0%) |
|  |  | **Mildly Abnormal** | 0 (0.0%) | 198 (100%) | 0 (0.0%) | 0 (0.0%) |
|  |  | **Moderately Abnormal** | 0 (0.0%) | 0 (0.0%) | 90 (78.3%) | 25 (21.7%) |
|  |  | **Severely Abnormal** | 0 (0.0%) | 0 (0.0%) | 0 (0.0%) | 80 (100%) |

# **Supplementary Table 3.** Agreement of LVEDDi partitions between 2006 and 2015 guidelines in men and women

: Re-classification into one higher grade

# **Supplementary table 4.** Factors related to the discrepancy between two LVEDDi classifications in guidelines of Chamber Quantification 2006 and Recommendations for Cardiac Chamber Quantification by Echocardiography in Adults 2015

| **Factor** | **B** | **Sig.** | **Exp(B) (95% CI)** |
| --- | --- | --- | --- |
|  |  |  |  |
| **Female sex** | -0.859 | 0.000 | 0.423 (0.332-0.539) |
| **Age** | -0.002 | 0.592 | 0.998 (0.992-1.005) |
| **Weight** | -0.033 | 0.000 | 0.968 (0.960-0.975) |
| **Height** | -0.038 | 0.000 | 0.963 (0.952-0.974) |
| **LVEF** | -0.039 | 0.000 | 0.961 (0.953-0.970) |
| **LV wall motion abnormality** |  | 0.083 |  |
| **No wall motion abnormality to global motion abnormality** | -0.120 | 0.601 | 0.887 (0.564-1.392) |
| **Regional motion abnormality to global motion abnormality** | 0.142 | 0.523 | 1.152 (0.746-1.780) |
| **Significant MR** | 0.763 | 0.000 | 2.144 (1.647-2.791) |
| **Significant AS** | 0.340 | 0.222 | 1.404 (0.815-2.421) |
| **Significant AR** | 0.914 | 0.000 | 2.493 (1.762-3.528) |

AS, aortic stenosis; AR, aortic regurgitation; LVEF, left ventricular ejection fraction; LV, left ventricle; LV, left ventricle; MR, mitral regurgitation
